# Quantitative Assessment of the Effect of Cytochrome P450 2C9 Gene Polymorphism and Colorectal Cancer

**DOI:** 10.1371/journal.pone.0060607

**Published:** 2013-04-05

**Authors:** Yuan Zhao, Yusong Han, Liang Zhang, Yichao Wang, Yushui Ma, Feng Zhang, Da Fu, Xiaofeng Wang

**Affiliations:** 1 Department of Gastroenterology, Zhongshan Hospital, Fudan University, Shanghai, People's Republic China; 2 Department of General Surgery, Zhongshan Hospital, Fudan University, Shanghai, People's Republic China; 3 Department of Orthopaedics Surgery, Zhongshan Hospital, Fudan University, Shanghai, People's Republic China; University of Texas School of Public Health, United States of America

## Abstract

CYP2C9 enzyme activity is involved in the metabolism of substances related to colorectal cancer (CRC), and it is functionally linked to a genetic polymorphism. Two allelic variants of the *CYP2C9* gene, namely CYP2C9*2 and CYP2C9*3, differ from wild-type CYP2C9*1 by single amino acid substitutions. These mutated alleles encode enzymes with altered properties that are associated with impaired metabolism. In the past decade, a number of case-control studies have been carried out to investigate the relationship between the *CYP2C9* polymorphism and CRC susceptibility, but the results were conflicting. To investigate this inconsistency, we performed a meta-analysis of 13 studies involving a total of 20,879 subjects for CYP2C9*2 and *3 polymorphisms to evaluate the effect of *CYP2C9* on genetic susceptibility for CRC. Overall, the summary odds ratio of CRC was 0.94 (95%CI: 0.87–1.03, P = 0.18) and 1.00 (95%CI: 0.86–1.16, P = 0.99) for *CYP2C9* *2 and *3 carriers, respectively. No significant results were observed in heterozygous and homozygous when compared with wild genotype for these polymorphisms. In the stratified analyses according to ethnicity, sample size, diagnostic criterion, HWE status and sex, no evidence of any gene-disease association was obtained. Our result suggest that the *2, *3 polymorphisms of *CYP2C9* gene are not associated with CRC susceptibility.

## Introduction

Colorectal cancer (CRC) is the third most common type of cancer in the western world and is responsible for approximately 50,000 deaths per year [1]. Family-based studies have suggested that the disease has a significant genetic component, with a large twin study conducted in Scandinavian countries suggesting that as many as 35% of colorectal cancers may be due to inherited susceptibility [2]. However, the recognized Mendelian predisposition syndromes, such as hereditary nonpolyposis colorectal cancer and adenomatus polyposis coli, account for less than 5% of the overall incidence of colorectal cancer [3]. Therefore, common, low-penetrance polymorphisms may confer a substantial part of the genetic risk, but given that the estimated effect of each polymorphism is expected to be small, large studies are necessary to reduce the size-related uncertainty of effects and provide robust evidence of association.

Specific components of the western diet including meat consumption (particularly red and/or well-done meat) and dietary fat (particularly polyunsaturated fatty acids) have been proposed as risk factors which influence susceptibility to colorectal cancer [4]–[5]. It has been suggested that this may be due to carcinogenic polycyclic aromatic hydrocarbons (PAH) and heterocyclic amines (HCA) produced when meat is cooked at high temperatures. Data from both in vitro and in vivo studies suggest that exposure to PAH significantly increase colorectal cancer risk [6], [7]. Cytochrome P450 2C9 (CYP2C9) is a key P450 enzyme which plays an important role in the metabolism and bioactivation of many dietary and environmental mutagens [8]. A variety of studies have demonstrated that the metabolism of PAH and other procarcinogens through CYP2C9 may well lead to the activation of the carcinogenic compounds [9], [10]. CYP2C9 enzyme activity in man is modulated by genetic polymorphisms. The variant alleles CYP2C9*2 (R144C) and *3 (I359L) produce slow-metabolizing enzymes compared with wild-type CYP2C9*1 [11], [12]. Hence, *CYP2C9* gene may be a good candidate for genetics studies on CRC.

Over the past few years, considerable efforts have been devoted to exploring the relationships between the *CYP2C9* polymorphisms and CRC risk among various populations. But the results are not always consistent. There are several possible explanations for this discordance, such as small sample size, ethnic background, uncorrected multiple hypothesis testing, and publication bias. Meta-analysis is a statistical procedure for combining the results of several studies to produce a single estimate of the major effect with enhanced precision. It has become important in cancer genetics because of rapid increases in the number and size of datasets. The aim of the present study is to perform a comprehensive meta-analysis to evaluate the association between the *CYP2C9* *2 and *3 polymorphism and CRC.

## Materials and Methods

### Literature search strategy

We searched the PubMed, Embase, and ISI Web of Science for all articles on the association between *CYP2C9* polymorphisms and CRC risk published before the end of May 2012. The following keywords were used: “colorectal” or “colo*,” “cancer” or “tumor” or “carcinoma,” and “*CYP2C9*” or “cytochrome P450 2C9”. Additional studies were identified by a hand search of references of original studies and review articles on the association between the *CYP2C9* polymorphism and CRC. No language restrictions were applied.

### Inclusion and exclusion criteria

We reviewed abstracts of all citations and retrieved studies. The following criteria were used to include published studies: (i) identification of colorectal cancer cases was confirmed histologically or pathologically, (ii) case–control or cohort studies to evaluate the association between *CYP2C9* *2 or *3 polymorphism and CRC risk and (iii) genotype distribution information in cases and controls or odds ratio (OR) with its 95% confidence interval (CI) and P-value. Major reasons for exclusion of studies were (i) review, or editorial, or comment; (ii) duplicated studies; (iii) no sufficient data were reported.

### Data abstraction

Two investigators extracted information from all eligible publications independently according to the inclusion criteria listed above. Disagreements were resolved by discussion with co-authors. For each included study, the following information was extracted from each report according to a fixed protocol: first author's surname, publication year, definition and numbers of cases and controls, diagnostic criterion, frequency of genotypes, source of controls, gender, age, Hardy–Weinberg equilibrium (HWE) status, ethnicity and genotyping method.

### Statistical methods

We first assessed HWE in the controls for each study using goodness-of-fit test (chi-square or Fisher's exact test) and a P<0.05 was considered as significant disequilibrium. The strength of the association between CRC and the *CYP2C9* *2 and *3 polymorphism was estimated using ORs, with the corresponding 95% CIs. For the *2 polymorphism, we first estimated the risks of the *2 heterozygous and *2 homozygote genotypes on CRC, compared with the wild-type *1 homozygote. The risk of *2 carrier versus *1 on cancers was then evaluated in dominant model. The same evaluation was carried out for the *3 polymorphism.

Both the Cochran's Q statistic [13] to test for heterogeneity and the I^2^ statistic to quantify the proportion of the total variation due to heterogeneity [14] were calculated. Random-effects and fixed-effect summary measures were calculated as inverse-variance-weighted average of the log odds ratio. The results of random-effects summary were reported in the text because it takes into account the variation between studies. The significance of the overall OR was determined by the Z-test. Subsidiary analyses included subgroup analyses or random-effects meta-regression with restricted maximum likelihood [15]. Ethnicity (Caucasian vs Asian), HWE status among control (yes or no), diagnostic criterion (ICD-9 vs. others), sample size (≥500 cases or <500 cases) and sex were pre-specified as characteristics for the assessment of heterogeneity. Ethnicity, sample size, HWE status, and sex distribution in cases and controls were analyzed as covariates in meta-regression.

In order to assess the stability of the results, one-way sensitivity analyses were performed by removing each individual study in turn from the total and re-analyzing the remainder. Egger's regression test and funnel-plot analysis were used to assess publication bias [16], [17]. Analyses were performed using the STATA software version 10.0 (Stata Corporation, College Station, TX, USA). All P values are two-sided at the P = 0.05 level.

## Results

### Characteristics of studies

There were 63 papers relevant to the searching terms. The study selection process is shown in Figure S1. A total of 13 studies examined the association between the *CYP2C9* polymorphism and CRC were included in the current meta-analysis [18]–[30]. Among them, 12 studies were identified for the *CYP2C9* *2 polymorphism, including a total of 9154 cases and 10900 controls, and for the *3 polymorphism 12 studies were identified covering a total of 7701 cases and 9287 controls. Characteristics of studies included in the current meta-analysis are presented in Table 1.

**Table 1:** Characteristics of the studies included in the meta-analysis.

Reference	Year	Ethnicity	Case	Control	No. of case	No. of control	Sex in case/control (male%)	MAF in controls		Genotyping method
								CYP2C9*2	CYP2C9*3	
Sainz [18]	2011	German	ICD-10	Healthy	1768	1783	58.6/59.8	0.12	0.06	KASPar assay
Cleary [19]	2010	Canadian	ICD-9	Healthy	1165	1292	41.0/56.0	0.14	0.07	Taqman
Northwood [20]	2010	British	Colonoscopy confirmed	Healthy	308	296	71.3/58.8	0.11	0.07	Taqman
Buyukdogan [21]	2009	Turkish	CRC patients	Healthy	77	78	52.0/53.0	0.11	0.05	RT-PCR
Cotterchio [22]	2008	Canadian	ICD-9	Healthy	834	1249	NA/NA	0.14	0.07	Taqman
Liao [23]	2007	Chinese	CRC patients	Healthy	284	483	54.5/53.6	/	0.03	Sequencing
Küry [24]	2007	French	CRC patients	Healthy	1013	1118	62.0/54.0	0.14	/	Taqman
Samowitz [25]	2006	American	ICD-9	Healthy	2295	2903	57.1/54.6	0.18	0.02	RFLP, TaqMan
McGreavey [26]	2005	British	ICD-9	Healthy	490	592	61.0/54.0	0.14	0.07	Taqman
Tranah [27]	2005	American	CRC patients	Healthy	416	825	0/0	0.17	0.08	Taqman
Landi [28]	2005	Spanish	CRC patients	Cancer free	364	324	NA/NA	0.13	0.08	Sequencing
Chan [29]	2004	American	Histology confirmed	Healthy	339	350	0/0	0.09	0.08	Taqman
Martínez [30]	2001	Spanish	Histology confirmed	Healthy	110	123	53.0/53.0	0.19	0.20	RFLP

ICD: International Classification of Diseases, HB: hospital-based, PB: population-based, MAF: minor allele frequency, NA: not available.

### CYP2C*2 and CRC risk

The pooled estimate for CRC risk of *CYP2C9* *2 polymorphism is shown in Figure? 1. The comparison between CRC cases and controls was investigated in 15 data sets. Overall, there was no significant heterogeneity among the studies concerning CRC risk of *CYP2C9* *2 polymorphism (P>0.05). The combined OR from the fixed-effects model was not significant with OR of 0.94 (95% CI: 0.87–1.03, P = 0.18) for the variant carriers. Similarly, no significant associations were found for heterozygous (OR = 0.92, 95% CI: 0.85–1.00, P = 0.06) and homozygous (OR = 1.19, 95% CI: 0.98–1.45, P = 0.07) when compared with wild genotype. Subgroup analysis for CRC risk of *CYP2C9* *2 was also performed to explore the sources of heterogeneity. In the subgroup analyses by ethnicity, sample size, HWE status, diagnostic criteria and sex, no significant results were found in almost all genetic models (Table 2).

**Figure 1:**
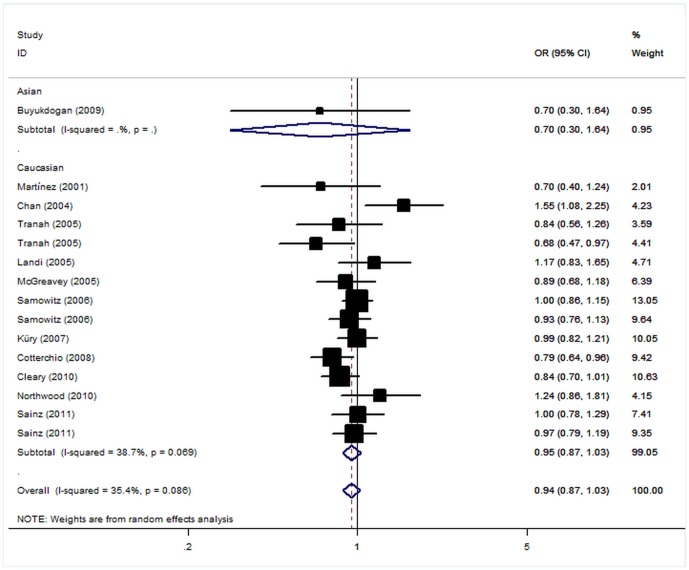
Forest plot (random effects model) of colorectal cancer risk associated with the CYP2C9 *2 carrier of the R144C polymorphism.

**Table 2:** Meta-analysis of the *CYP2C9* *2 polymorphism on colorectal cancer susceptibility.

Sub-group analysis	No. of data sets	No. of case/control	Heterozygous				Homozygous				Variant carrier			
			OR (95%CI)	P(Z)	P(Q)	I^2^	OR (95%CI)	P(Z)	P(Q)	I^2^	OR (95%CI)	P(Z)	P(Q)	I^2^
Total	15	9154/10900	0.92 (0.85–1.00)	0.06	0.12	31%	1.19 (0.98–1.45)	0.07	0.50	0%	0.94 (0.87–1.03)	0.18	0.09	35%
Ethnicity														
Caucasian	14	9077/10822	0.92 (0.85–1.01)	0.07	0.09	35%	1.20 (0.99–1.46)	0.06	0.46	0%	0.95 (0.87–1.03)	0.21	0.07	39%
Asian	1	77/78	0.72 (0.28–1.82)	0.48	NA	NA	0.64 (0.10–3.94)	0.63	NA	NA	0.70 (0.30–1.64)	0.41	NA	NA
Sample size														
<500	8	2093/2566	0.93 (0.76–1.14)	0.50	0.06	48%	1.40 (0.86–2.29)	0.18	0.18	31%	0.97 (0.78–1.20)	0.77	0.03	55%
≥500	7	7061/8334	0.92 (0.85–0.99)	0.03	0.35	10%	1.13 (0.91–1.42)	0.27	0.88	0%	0.93 (0.87–1.00)	0.05	0.48	0%
HWE status														
Yes	14	9077/10822	0.92 (0.85–1.01)	0.07	0.09	35%	1.20 (0.99–1.46)	0.06	0.46	0%	0.95 (0.87–1.03)	0.21	0.07	39%
No	1	77/78	0.72 (0.28–1.82)	0.48	NA	NA	0.64 (0.10–3.94)	0.63	NA	NA	0.70 (0.30–1.64)	0.41	NA	NA
Diagnostic criterion														
ICD criterion	7	6538/7808	0.90 (0.83–0.98)	0.01	0.38	6%	1.11 (0.88–1.40)	0.37	0.92	0%	0.92 (0.85–0.99)	0.03	0.54	0%
Other criterion	8	2616/3092	0.95 (0.79–1.15)	0.62	0.07	47%	1.44 (0.90–2.29)	0.13	0.19	30%	0.99 (0.81–1.20)	0.92	0.03	54%
Sex														
Male	2	1273/1455	0.79 (0.52–1.18)	0.25	0.06	71%	1.27 (0.77–2.11)	0.35	0.60	0%	0.83 (0.59–1.18)	0.31	0.09	66%
Female	3	1238/1492	1.11 (0.85–1.44)	0.46	0.17	44%	0.92 (0.34–2.45)	0.86	0.10	57%	1.09 (0.79–1.51)	0.60	0.06	64%

NA: not available.

Although the formal test for heterogeneity was not significant, we conducted meta-regression as there were also grounds for considering the ethnicity, sample size, HWE status, and sex distribution among cases and controls as potential sources of heterogeneity. However, the meta-regression showed that none of these covariates significantly contributed to the heterogeneity among the individual study results (P>0.05 for all).

### CYP2C9 *3 and CRC risk

**Figure 2:**
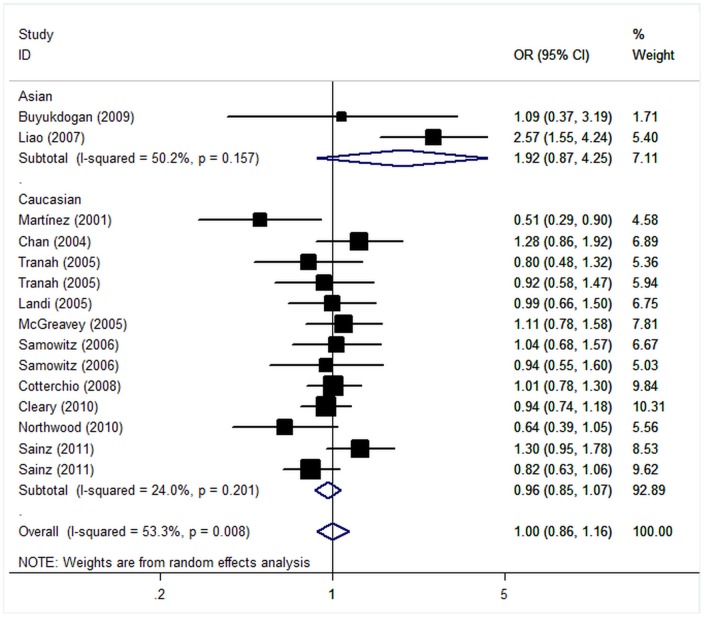
Forest plot (random effects model) of colorectal cancer risk associated with the CYP2C9 *3 carrier of the I359L polymorphism.

Overall, the variant genotypes of the *CYP2C9* *3 were not associated with CRC risk when compared with the wild-type *1 homozygote (*3 heterozygous: OR = 1.00, 95% CI = 0.86–1.17, P = 0.99; *3 homozygous: OR = 0.87, 95% CI = 0.43–1.74, P = 0.69). Similarly, no associations were observed in the dominant genetic model (OR = 1.00, 95% CI = 0.86–1.16, P = 0.99; Figure 2). On the basis of the potential underestimation of the true effect of the polymorphism on the CRC risk, we stratified these studies according to ethnicity, sample size, HWE status, diagnostic criteria and sex. In stratified analyses, the variant genotypes had no significant relationship with CRC in all of the subgroups, compared with wild-type. Similar results were observed in the dominant genetic model (Table 3)

**Table 3:** Meta-analysis of the *CYP2C9* *3 polymorphism on colorectal cancer susceptibility.

Sub-group analysis	No. of data sets	No. of case/control	Heterozygous				Homozygous				Variant carrier			
			OR (95%CI)	P(Z)	P(Q)	I^2^	OR (95%CI)	P(Z)	P(Q)	I^2^	OR (95%CI)	P(Z)	P(Q)	I^2^
Total	15	7701/9287	1.00 (0.86–1.17)	0.99	0.007	54%	0.87 (0.43–1.74)	0.69	0.32	14%	1.00 (0.86–1.16)	0.99	0.008	53%
Ethnicity														
Caucasian	13	7343/8734	0.96 (0.85–1.08)	0.49	0.18	26%	0.87 (0.43–1.74)	0.69	0.32	14%	0.96 (0.85–1.07)	0.45	0.20	24%
Asian	2	358/553	1.92 (0.87–4.25)	0.11	0.16	50%	NA	NA	NA	NA	1.92 (0.87–4.25)	0.11	0.16	50%
Sample size														
<500	9	2314/2933	0.99 (0.74–1.33)	0.95	0.001	69%	1.44 (0.49–4.19)	0.51	0.56	0%	1.00 (0.75–1.33)	0.99	0.002	68%
≥500	6	5387/6354	1.00 (0.88–1.13)	0.97	0.46	0%	0.65 (0.25–1.66)	0.37	0.23	27%	0.98 (0.87–1.11)	0.76	0.40	2%
HWE status														
Yes	13	7443/8786	1.05 (0.90–1.22)	0.52	0.02	49%	0.87 (0.43–1.74)	0.69	0.32	14%	1.05 (0.90–1.21)	0.54	0.02	49%
No	2	258/521	0.65 (0.42–1.01)	0.05	0.25	25%	NA	NA	NA	NA	0.65 (0.42–1.01)	0.05	0.25	25%
Diagnostic criterion														
ICD criterion	7	5877/6946	1.01 (0.90–1.14)	0.85	0.53	0%	0.66 (0.31–1.38)	0.27	0.33	13%	0.99 (0.89–1.11)	0.91	0.48	0%
Other criterion	8	1824/2341	0.97 (0.69–1.36)	0.85	0.0007	72%	3.32 (0.67–16.49)	0.14	0.92	0%	0.98 (0.70–1.38)	0.92	0.001	71%
Sex														
Male	2	1257/1391	0.84 (0.67–1.06)	0.15	0.66	0%	0.67 (0.11–4.02)	0.66	NA	NA	0.84 (0.67–1.06)	0.14	0.65	0%
Female	3	1199/1437	1.14 (0.88–1.49)	0.32	0.27	24%	2.10 (0.52–8.43)	0.29	0.94	0%	1.15 (0.88–1.52)	0.30	0.25	29%

NA: not available.

In meta-regression analysis, sample size (p = 0.93), gender of cases (P = 0.34) and controls (P = 0.37), diagnostic criteria (P = 0.91) and the status of Hardy–Weinberg equilibrium (p = 0.05) did not significantly explained such heterogeneity. By contrast, ethnicity (P = 0.001) were significantly correlated with the magnitude of the genetic effect.

### Sensitivity analyses and Publication bias

A single study involved in the meta-analysis was deleted each time to reflect the influence of the individual dataset to the pooled ORs, and the corresponding pooled ORs were not qualitatively altered, suggesting that the results of this meta-analysis are stable (Figure S2 and S3). In addition, when excluding the studies that were not in HWE, the results were persistent and robust (Table 2 and 3). The shape of the funnel plot did not indicate any evidence of obvious asymmetry (Figure S4 and S5), thus suggesting no publication bias among the studies included. The statistical results still did not show preferential publication of positive findings in smaller studies (Egger test, P = 0.96 for *2 carrier; P = 0.95 for *3 carrier).

## Discussion

A number of factors predict CRC; however, detailed mechanisms of CRC remain a matter of speculation. However, accumulative evidences have suggested an important role for genetics in determining risk for CRC [2]. Association studies are appropriate for searching susceptibility genes involved in CRC [31]. CYP2C9 is a key P450 enzyme implicated in the metabolism of exogenous and endogenous substrates. A variety of studies have demonstrated that the metabolism of polycyclic aromatic hydrocarbons and other procarcinogens through CYP2C9 may well lead to the activation of the carcinogenic compounds [19], [27]. *CYP2C9* is a polymorphic gene in the human population; the genetic variations have been studied with respect to a variety of cancer types. In recent years, a number of molecular epidemiologic studies have been conducted to evaluate the role of *2 and *3 polymorphisms in the *CYP2C9* gene on CRC risk; however, the results remain conflicting rather than conclusive. As a powerful statistical method, meta-analysis can provide a quantitative approach for pooling the results of different research on the same topic, and thus a quantitative assessment of association between *CYP2C9* polymorphism and CRC risk was of great value. The present meta-analysis, including 9463 cases and 11416 controls from 13 case–control studies, explored the association between the *2 and *3 polymorphism of *CYP2C9* gene and CRC risk. To the best of our knowledge, this is the first comprehensive meta-analysis concerning the relationship between *CYP2C9* polymorphism and CRC susceptibility. Overall, we did not find any significant association between *CYP2C9* *2, *3 polymorphism and CRC susceptibility. In the stratified analysis by ethnicity, sample size, HWE status, diagnostic criteria and sex, significant associations were still not observed in all genetic models. *CYP2C9* genotype was not found to be a determinant of colorectal cancer susceptibility according to the results of present meta-analysis.

Although CYP2C9 is active in the metabolism of several commonly prescribed drugs, including warfarin, phenytoin and non-steroidal anti-inflammatory drugs (NSAIDs) [32], its role in xenobiotic and carcinogen metabolism is less well defined, although it has been shown to metabolize the carcinogen benzo[a]pyrene (B[a]P) to the highly mutagenic metabolite B[a]P-7,8diol-9,10-epoxide [33]. CYP2C9*3 encodes a protein with approximately 5–30% of the activity of the common reference allele [34], and it could therefore be hypothesized that CYP2C9*3 allele carriers have reduced carcinogen activating ability and thus reduced disease risk. However, the increased CRC risk associated with CYP2C9*2 genotype in earlier studies suggests that the enzyme may play a more important role in detoxification of carcinogens.

Epidemiological data indicated the use of NSAID is inversely associated with the risk of developing colorectal cancer [35]. Metabolism of NSAIDs involves oxidation by CYP enzymes and/or conjugation, particularly glucuronidation by phase II enzymes. The major enzymes involved are CYP2C9 and UGT1A6, both enzymes have variant forms [26]. The modulation of chemoprevention of NSAIDs on CRC risk by the genotype of *CYP2C9* and *UGT1A6* has been reported among Caucasian population [26], [36]. Recently, the interaction between *UGT1A6* and *CYP2C9*, aspirin or ibuprofen use, and CRC risk were determined in 2295 CRC cases and 2903 controls. Their data showed the enhanced effect of slower-metabolizing *CYP2C9* variants on the chemopreventive activity of ibuprofen against CRC, and CYP variants were more effective in individuals with wild-type rather than variant *UGT1A6* [25]. Therefore, the contribution of gene – gene interaction as well as gene – medication interaction should be considered in future study in elucidating the contribution of *CYP2C9* polymorphism to CRC etiology.

Although there is no association between *CYP2C9* *2, *3 polymorphism and the risk of CRC, the associations between *CYP2C9* polymorphism and CRC risk might be modified when exposed to some factors such as tobacco smoking [29]. Chan et al [29] reported that *CYP2C9* genotypes modified the CRC risk associated with smoking status. Women with variant genotypes who smoked >20 pack-years had a 2.5-fold greater odds of adenoma compared with women with wild-type genotypes who smoked ≤20 pack-years. Because of limited data in primary studies, we could not quantitatively analyze the modification effects of smoking on the relationships between *CYP2C9* polymorphisms and CRC risk.

Limitations also inevitably existed in this meta-analysis. Firstly, most studies were conducted in Caucasian population. Therefore, it could be under powered to detected the interaction among Asian population in subgroup analysis. Hence, further studies including a wider spectrum of subjects should be carried to investigate the role of these variants in different populations. Secondly, our results were based on unadjusted estimates since we did not have original data. Therefore, we were not able to take into account other factors like obesity, inflammation, aspirin/NSAID use, vitamin D and vitamin E intake, which may modify the risk estimates, as reported in previous publications. Thus, assessment of the association between *CYP2C9* polymorphism and these covariates and CRC is needed in order to determine clearly the impact of *CYP2C9* polymorphism on the etiology of CRC. Thirdly, meta-analysis is a type of retrospective study, and the recall and selection bias might exist. In spite of these, our meta-analysis also had some advantages. First, substantial numbers of cases and controls were pooled from different studies, which significantly increased the statistical power of the analysis. Second, no publication bias was detected; indicating that the whole pooled result may be unbiased.

In conclusion, this meta-analysis evaluates the relation-ship between genetic polymorphism and CRC risk and reveals that *CYP2C9* *2 and *3 polymorphism is not associated with altered susceptibility to CRC. As studies among other populations are currently limited, further studies including a wider spectrum of subjects should be carried to investigate the role of these variant in these populations, which should lead to better, comprehensive understanding of the association between the *CYP2C9* polymorphism and CRC.

This meta-analysis is guided by the PRISMA statement (Checklist S1).

## Supporting Information

Figure S1 **The association study selection process.**
(TIF)

Figure S2 Result of sensitivity analyses for CYP2C9*2 carrier and CRC risk.
(TIF)

Figure S3 Result of sensitivity analyses for CYP2C9*3 carrier and CRC risk.
(TIF)

Figure S4 Funnel plot for the association between and CYP2C9 *2 carrier and colorectal cancer risk; Egger's test was also performed to investigate the symmetry of the funnel plot ( ***P*** **= 0.96).**
(TIF)

Figure S5 Funnel plot for the association between and CYP2C9 *3 carrier and colorectal cancer risk; Egger's test was also performed to investigate the symmetry of the funnel plot ( ***P*** **= 0.95).**
(TIF)

Checklist S1 **PRISMA 2009 Checklist.**
(DOC)
