# Supplementary figures and images for: Quantitative Assessment of the Effect of Cytochrome P450 2C9 Gene Polymorphism and Colorectal Cancer
Source: PLoS One. 2013 Apr 5;8(4):e60607. doi: 10.1371/journal.pone.0060607 (PMC3618415; doi:10.1371/journal.pone.0060607)

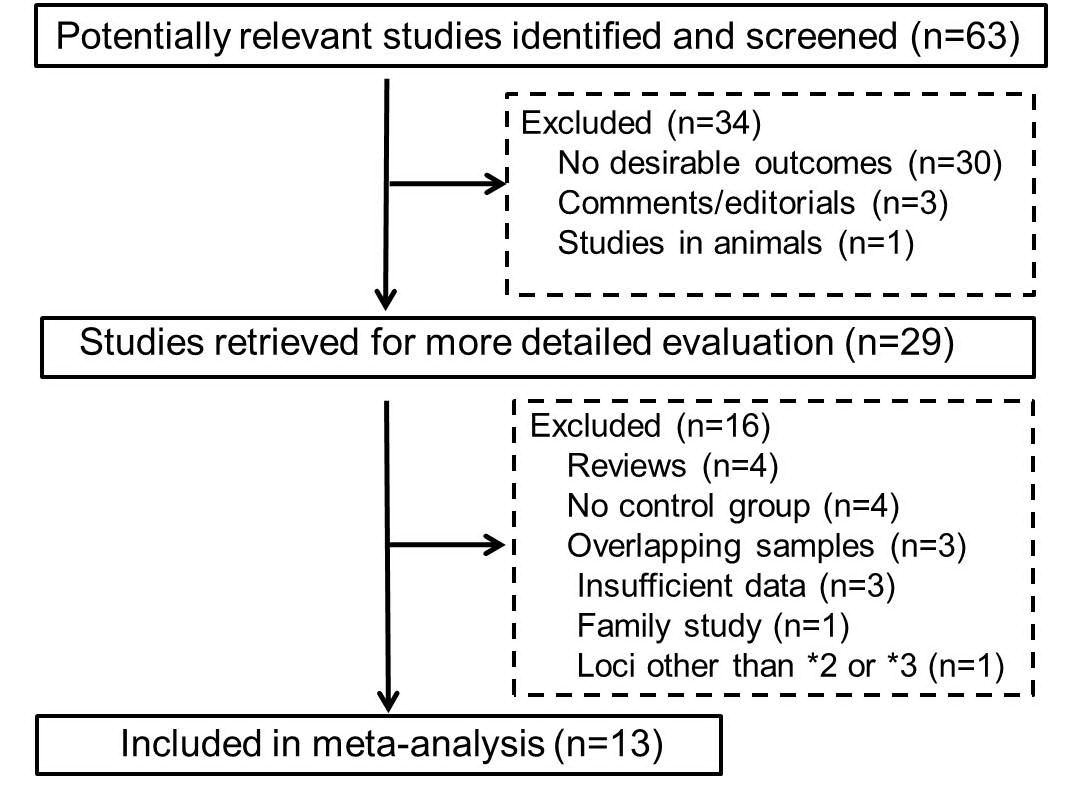

Supplement: Figure S1 — The association study selection process. (TIF) [file pone.0060607.s001.tif]

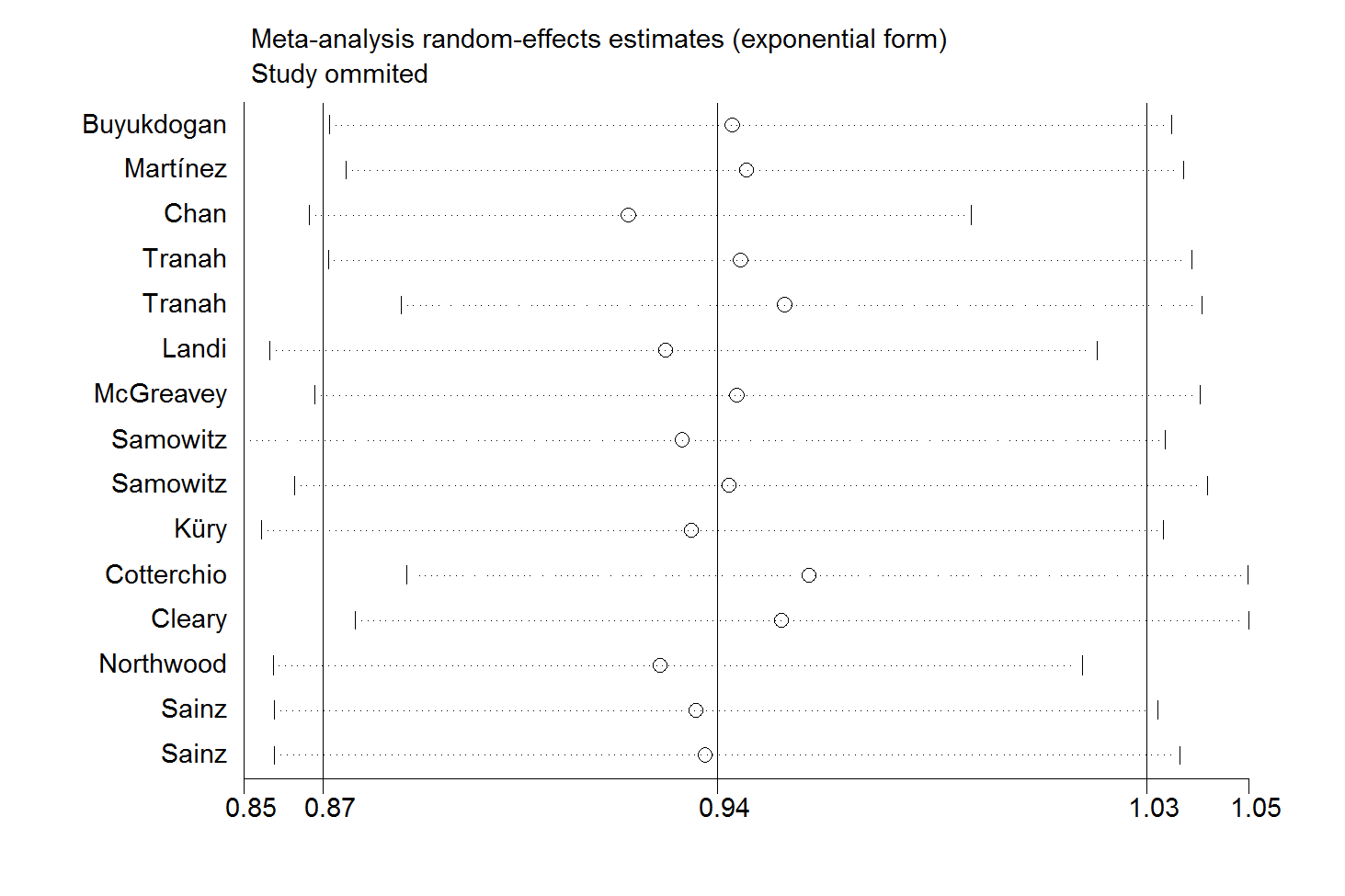

Supplement: Figure S2 — Result of sensitivity analyses for CYP2C9*2 carrier and CRC risk. (TIF) [file pone.0060607.s002.tif]

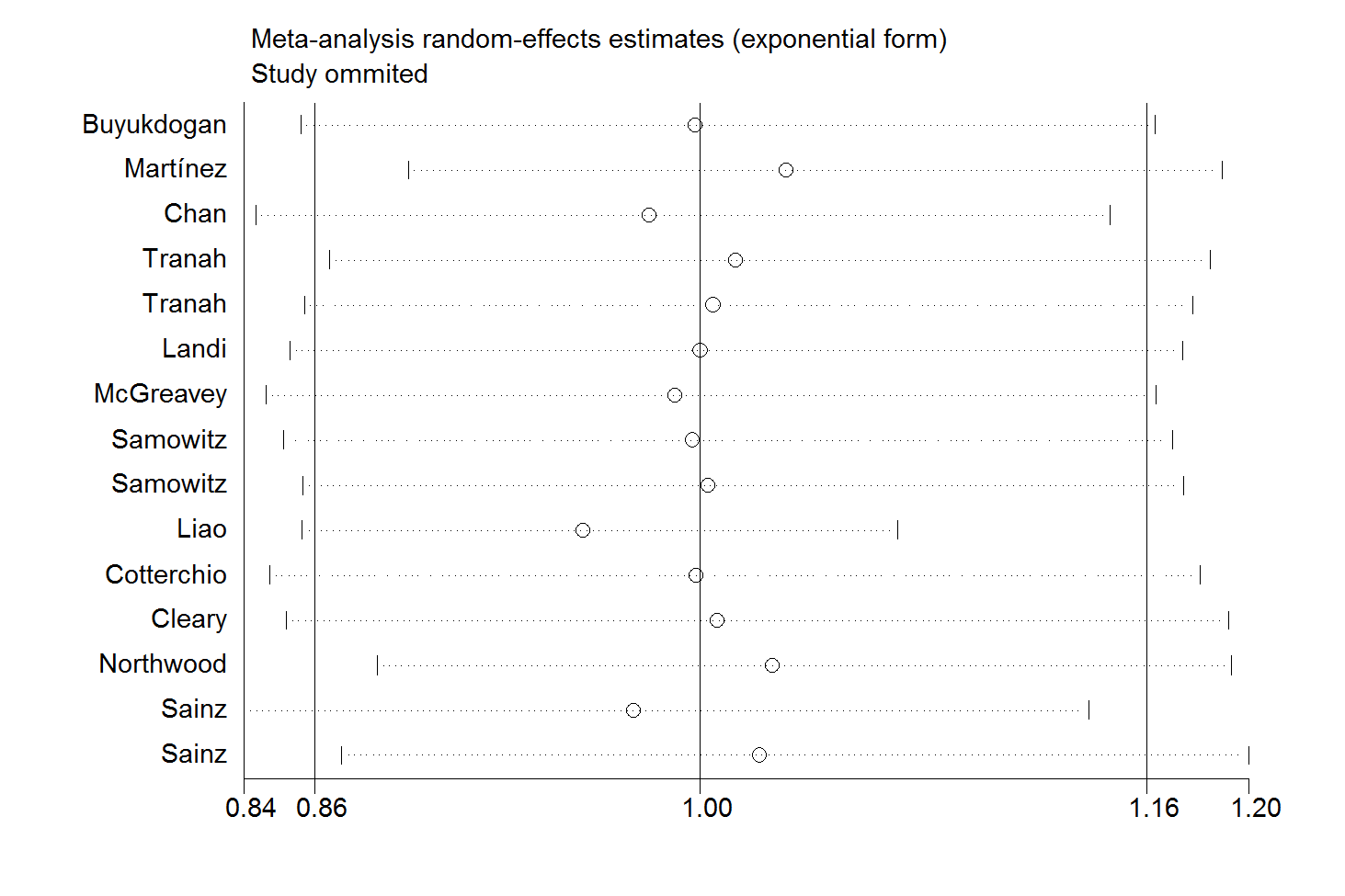

Supplement: Figure S3 — Result of sensitivity analyses for CYP2C9*3 carrier and CRC risk. (TIF) [file pone.0060607.s003.tif]

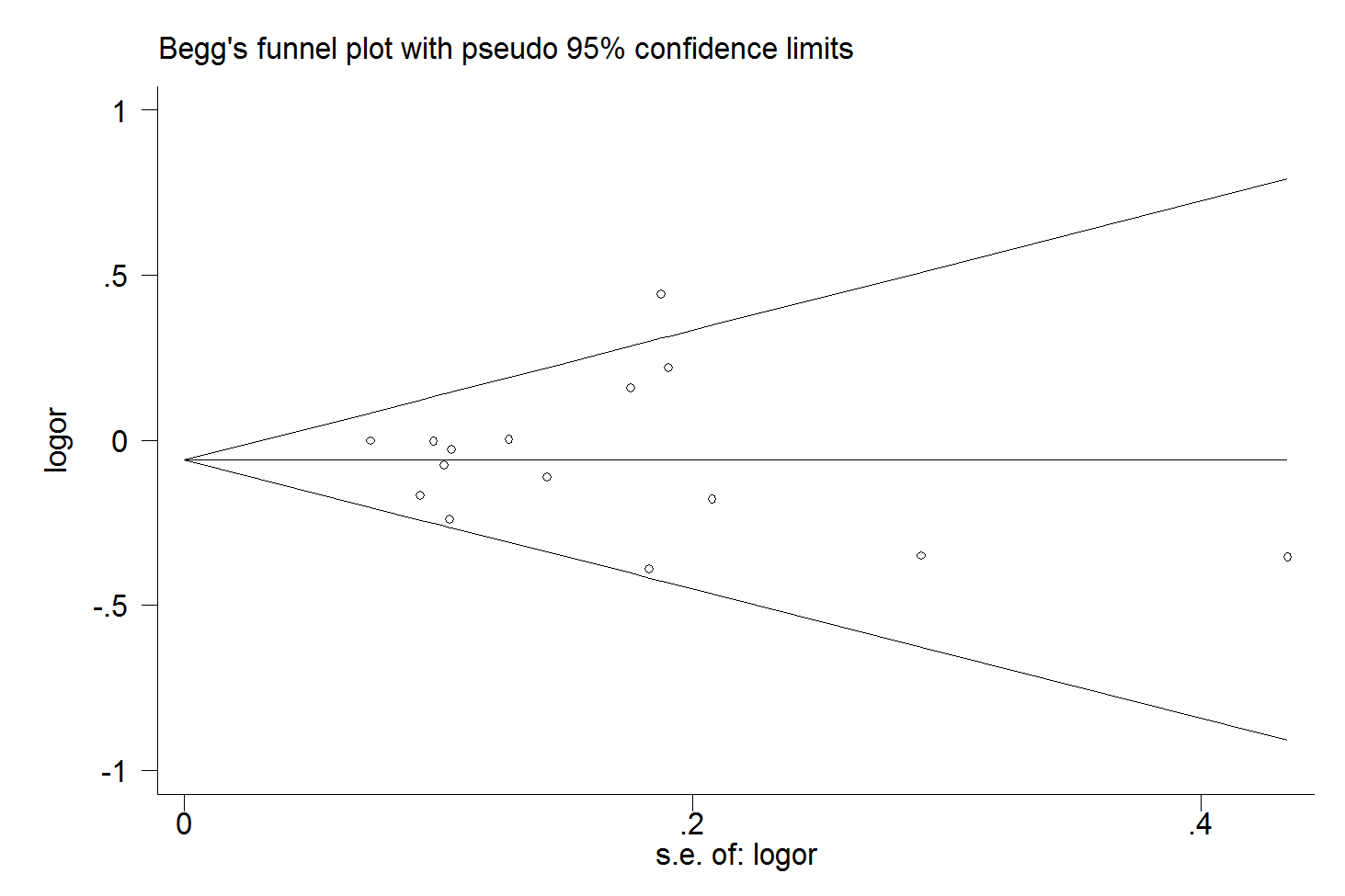

Supplement: Figure S4 — Funnel plot for the association between and CYP2C9 *2 carrier and colorectal cancer risk; Egger's test was also performed to investigate the symmetry of the funnel plot ( P = 0.96). (TIF) [file pone.0060607.s004.tif]

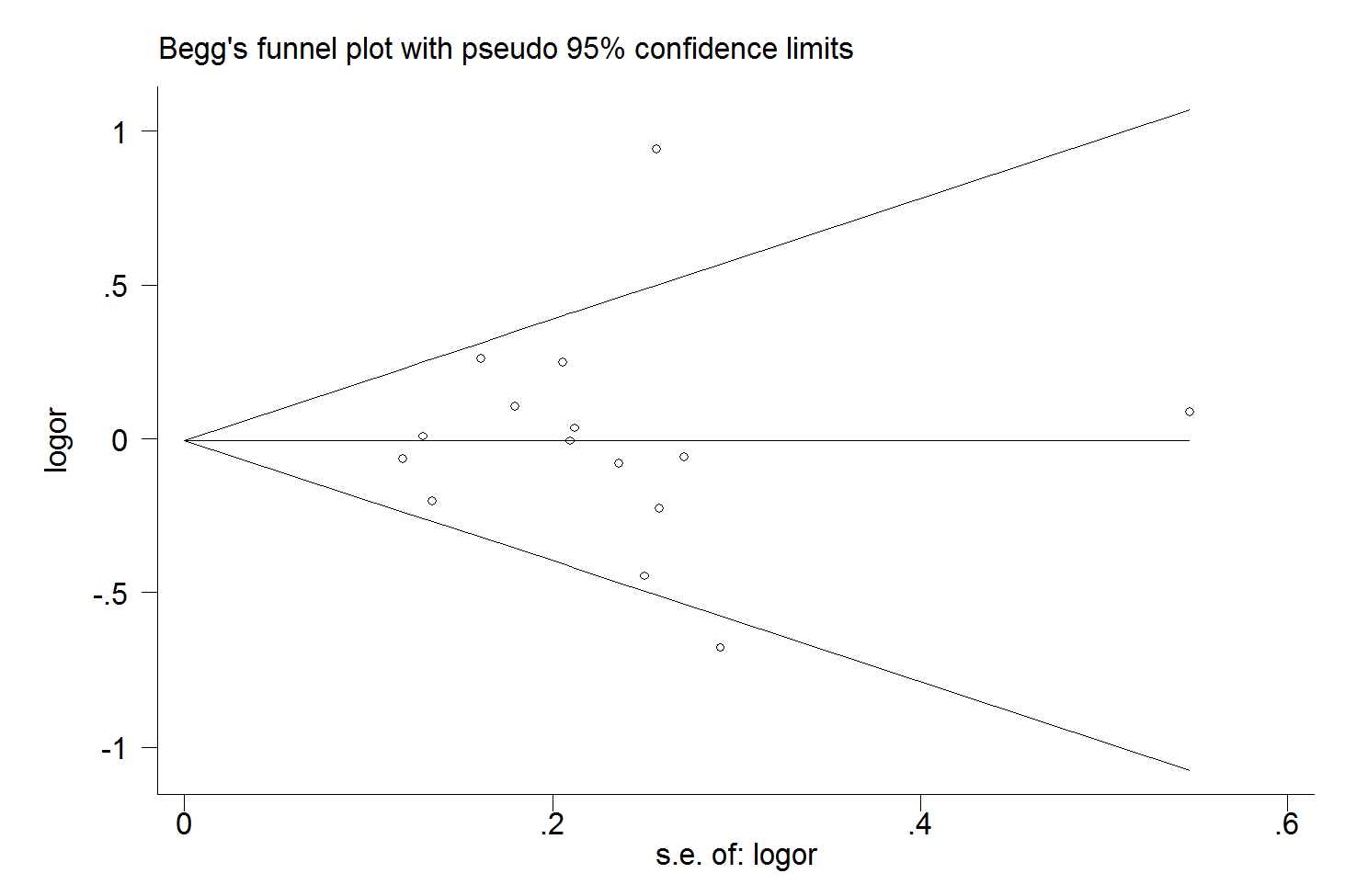

Supplement: Figure S5 — Funnel plot for the association between and CYP2C9 *3 carrier and colorectal cancer risk; Egger's test was also performed to investigate the symmetry of the funnel plot ( P = 0.95). (TIF) [file pone.0060607.s005.tif]
